# A New Approach to Deliver Anti-cancer Nanodrugs with Reduced Off-target Toxicities and Improved Efficiency by Temporarily Blunting the Reticuloendothelial System with Intralipid

**DOI:** 10.1038/s41598-017-16293-6

**Published:** 2017-11-23

**Authors:** Li Liu, Qing Ye, Maggie Lu, Shih-Ta Chen, Hsiang-Wen Tseng, Ya-Chin Lo, Chien Ho

**Affiliations:** 1 Department of Biological Sciences, Carnegie Mellon University, Pittsburgh, PA USA; 2 Drug Delivery Technology Department, Targeted Drug and Delivery Technology Division, Biomedical Technology and Device Research Laboratories, Industrial Technology Research Institute, Hsinchu, Taiwan; 3 Present Address: National Institute of Neurological Disorders and Stroke, National Institute of Health, Bethesda, MD USA; 4 Present Address: Department of Neurology, University of Pittsburgh School of Medicine, Pittsburgh, PA USA

## Abstract

We have developed a new strategy to temporarily blunt the reticuloendothelial system uptake of nanodrugs, a major challenge for nanodrug delivery and causing off-target toxicities, using an FDA approved nutrition supplement, Intralipid. We have tested our methodology in rats using an experimental platinum-containing anti-cancer nanodrug and three FDA approved nanodrugs, Abraxane, Marqibo, and Onivyde, to determine their toxicities in liver, spleen, and kidney, with and without the addition of Intralipid. Our method illustrates its potentials to deliver nanodrugs with an increase in the bioavailability and a decrease in toxicities. Our study shows that Intralipid treatment exhibits no harmful effect on tumor growing and no negative effect on the anti-tumor efficacy of the platinum-containing nanodrug, as well as animal survival rate in a HT-29 xenograft mouse model. Our methodology could also be a valuable complement/supplement to the “stealth” strategies. Our approach is a general one applicable to any approved and in-development nanodrugs without additional modification of the nanodrugs, thus facilitating its translation to clinical settings.

## Introduction

Nanoparticle-based anti-cancer drugs have been developed over the past three decades for treatment of different types of cancer^1–3^. Seven anti-cancer nanodrugs, i.e., Abraxane, DaunoXome, DepoCyt, Doxil, Marqubio, Oncaspar, and Onivyde, have been approved by the Food and Drug Administration (FDA). Over thirty nanodrugs are under current clinical trial investigations. Major advantages for nanodrugs, i.e., nanoformulation of conventional anti-cancer drugs, are to enhance their delivery to tumor sites through the enhanced permeability and retention (EPR) effect^4–6^, reduce the toxicity of the drugs to non-targeting organs, and enhance pharmaceutical properties (e.g., bioavailability, solubility, and stability)^1–3^. Nanodrugs have the potential to provide more effective and safer treatment for cancer patients.

A major challenge for the above-mentioned advantages and potentials of nanodrugs is their rapid uptake by the reticuloendothelial system (RES), especially by the liver and spleen^1,3,7–9^. After a systemic administration, nanodrugs are rapidly taken up by the phagocytic cells in the RES^10^. RES organs are the major ones competing with tumors for the delivery of anti-cancer nanodrugs^1,7–9^. This off-targeting accumulation not only decreases the bioavailability and tumor delivery of the nanodrugs, but also increases their toxic side effects to patients^7,11–13^.

Strategies to decrease the RES uptake of the nanodrugs can improve their tumor targeting and also decrease their off-target side effects. To achieve this goal, many studies have been conducted to modify the nanoparticle characteristics and surface properties, such as size, shape, charge, composition, tumor targeting moiety. Polyethylene glycol (PEG)^9,14–18^ is the most commonly used for the surface modification. However, the present status of using nanodrugs is that a very small fraction (0.7%, median) of the injected nanodrugs is delivered to tumors, as summarized by a recent analysis of the literature data during the past 10 years^7^. Moreover, from a toxicological perspective, the complexity of the nanoparticles can be counter-productive and also each additional modification could cause an extra risk of toxicity^13^. The RES uptake and the resulting toxicity are major challenges for the development and the clinical translation of nanodrugs, e.g., siRNA-based nanodrugs^12–14^.

The accumulation and the side effects in the RES have also been reported by the FDA on its approved nanodrugs, including the successful ones [e.g., Abraxane^19,20^ and Doxil/Caelyx^21^] and the recently approved anti-cancer nanodrugs [e.g., Marqibo^22^ and Onivyde^23–25^]. The side effects, e.g., discomfort, nausea, and vomiting, are related to damages at the liver and the spleen. It is a big challenge to modify these approved drugs to reduce RES uptake and toxicity, because each new modification calls for additional efficacy, toxicity, and pharmacology studies before translating them to a clinical setting.

We have developed a novel strategy to temporarily blunt the RES uptake of nanoparticles, thus also can reduce the RES toxicity, by using an FDA approved nutritional supplement, Intralipid^26–28^. Intralipid has been safely used in the clinics as a source of parenteral nutrition for over three decades^29^. Kupffer cells in the liver are known to play an important role in the uptake and metabolism of Intralipid^30^. Intralipid infusion has been reported to inhibit RES function by possibly inhibiting peritoneal clearance and impairing the phagocytic activity of Kupffer cells^31^. Our hypothesis is that the uptake of nanodrugs by the RES can be reduced by using agents, such as Intralipid, which are also taken up by the Kupffer cells, i.e., competing with the nanodrugs, thus reducing the amount of drugs delivered to RES and also their toxicity in the RES organs.

We have first tested our new strategy by using nano- and micron-sized superparamagnetic iron-oxide (SPIO) particles^26^ [used as magnetic resonance imaging (MRI) contrast agents] and an in-development platinum (Pt)-containing anti-cancer nanodrug, dichloro (1,2-diaminocyclohexane) platinum (II)-loaded with hyaluronic acid polymer-coated nanodrug (DACHPt/HANP)^27^. In our “proof-of-concept” studies, rats were treated with a single dose of Intralipid 20% [2 g/kg, clinical dosage; intravenously (i.v.), clinical route] one hour before the i.v. injection of the superparamagnetic iron-oxide nano- or micon-sized particles. This treatment can result in a ~50% decrease in liver uptake and a ~3-fold increase in blood half-life of nano- and micron-sized iron-oxide particles. With respect to DACHPt/HANP, the Intralipid 20% treatment was found to reduce Pt accumulation in the liver, spleen, and kidney by 20 to 40% at 24-hr post nanodrug administration. The bioavailability of the Pt-nanodrug increases by 18.7% and 9.4% during the first 5 and 24 hr, respectively. These data are summarized in Table 1. Moreover, Intralipid treatment can significantly reduce the toxicities of DACHPt/HANP in the liver, spleen, and, notably, in the kidney^27^.

**Table 1:** Intralipid changes the biodistribution and bioavailability of an experimental Pt-containing anti-cancer nanodrug, DACHPt/HANP*.

DACHPt/HANP	Biodistribution									Bioavailability (ppm*min)**		
	Liver (μg Pt/g)			Spleen (μg Pt/g)			Kidney (μg Pt/g)			5 hr	24 hr	72 hr
	5 hr	24 hr	72 hr	5 hr	24 hr	72 hr	5 hr	24 hr	72 hr			
No Intralipid	8.6 ± 0.6	18.1 ± 2.2	10.1 ± 1.6	6.9 ± 1.2	26.2 ± 2.5	16.9 ± 2.9	4.9 ± 0.3	6.1 ± 1.5	7.9 ± 1.4	2610 ± 115	8053 ± 289	9336 ± 274
Intralipid Pre-injection	6.6 ± 0.5	13.9 ± 1.6	11.8 ± 3.7	4.2 ± 0.6	15.3 ± 1.2	7.3 ± 1.6	3.2 ± 0.5	4.2 ± 0.2	5.9 ± 0.7	3099 ± 180	8816 ± 401	9709 ± 126
% Changes Upon Intralipid Treatment***	↓24.9 p < 0.001	↓20.4 p < 0.001	↑8.7 p > 0.1	↓40.1 p < 0.001	↓42.4 p < 0.001	↓67.2 p < 0.001	↓34.0 p < 0.001	↓31.2 p < 0.001	↓28.7 p < 0.01	↑18.7 p < 0.001	↑9.4 p < 0.01	No Change

*Table 1 is modified from Figs 6 and 7 of ref.^27^, with permission. In this experiment, a single dose of Intralipid was administrated intravenously (i.v.) 1 hr before i.v. injection of DACHPt/HANP (2 mg Pt/kg). At 5-, 24-, and 72-hr post injection of DACHPt/HANP, tissues (liver, spleen, and kidney) were collected for the Pt-level determination. Blood was sampled after DACHPt/HANP injection at 1, 5, 10, 20, 45, and 60 min, 3, 5, 24, 28, 48, 52, and 72 hr. **Bioavailability was calculated by the area under the curve (AUC), namely the integral of the Pt concentration-time curve, using the trapezoidal rule with the use of KaleidaGraph 4.1 (Synergy Software, Reading, PA). ***% Changes for biodistribution are calculated from the total amount of Pt-drug accumulated in the organs. Statistical analysis was carried out with the Student’s *t* test. A p value < 0.05 was considered statistically significant.

The present study is an extension of our previous results. First, we have tested the effects of multi-doses of Intralipid 20% to deliver multi-doses of DACHPt/HANP in our rat model. Second, we have also tested our Intralipid methodology to deliver three FDA-approved anti-cancer nanodrugs, namely, Abraxane (paclitaxel albumin-stabilized nanoparticle formulation), Marqibo (vincristine sulfate liposome injection), and Onivyde or MM-39 (a prodrug, irinotecan liposome injection). We have found that Intralipid 20% treatment can significantly reduce the toxic side effects of all the four nanodrugs, especially in the spleen and kidney. Our positive findings suggest that our Intralipid methodology could become a new way to deliver anti-cancer nanodrugs as well as other nanodrugs, thus will encourage additional animal experiments and clinical studies to design the optimal way to deliver anti-cancer and other nanodrugs using Intralipid. It can also be used as a complement/supplement to various “stealth” strategies to deliver nanodrugs. A unique feature of our methodology is that we do not need to make any modifications on the existing FDA approved nanodrugs and their carriers. This feature will facilitate clinical translation of our methodology.

## Results

### Intralipid Reduces Toxic Side Effects of DACHPt/HANP

**Figure 1:**
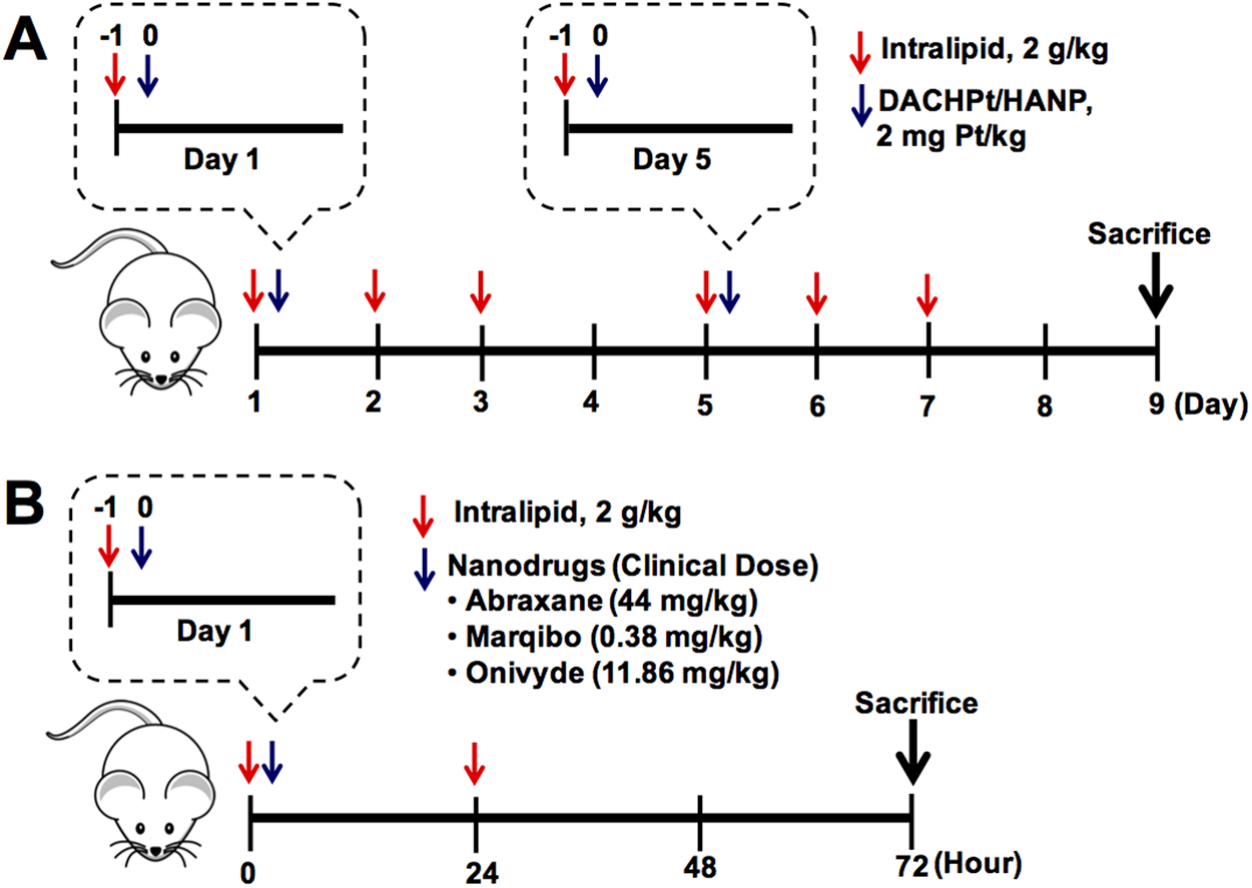
Treatment protocols for animal experiments. (**A**) Treatment protocol for using multiple-doses of Intralipid 20% to investigate the delivery of two doses of DACHPt/HANP. Rats (N = 6 for each group, i.e., Intralipid-treated group and control group) were treated with DACHPt/HANP (2 mg Pt/kg) intravenously twice a week (on Days 1 and 5). Also on Days 1 and 5, Intralipid was intravenously administered at a dosage of 2 g/kg 1 hr before the administration of the nanodrug. On Days 2, 3, 6, and 7, the rats were administered 2 g/kg of Intralipid 20% each day. (**B**) Treatment protocol for using Intralipid to investigate the delivery of three FDA-approved nanodrugs, Abraxane, Marqibo, and Onivyde. Intralipid was administered intravenously at 2 g/kg 1 hr before the treatment with the nanodrug. After 1 hr, Abraxane, Marqibo, or Onivyde was administered intravenously to a rat at the clinical dose (44 mg/kg, 0.38 mg/kg, and 11.86 mg/kg, respectively). The second dose of Intralipid (2 g/kg) was administered 24-hr post nanodrug treatment. For Abraxane experiment, N = 4 for each group, i.e., the Intralipid-treated group and the control group; for Marqibo and Onivyde experiment, N = 6 for each group. In all the animal experiments, PBS was administered to the control animals.

Our previous study^27^ was designed as a “proof-of-concept” study to show that Intralipid 20% pre-treatment can change the RES uptake, bioavailability, and toxicological profiles of DACHPt/HANP. These results are summarized in Table 1, which shows that its uptake in the liver, spleen, and kidney diminishes and that its bioavailability increases when Intralipid is administered 1 hr before intravenous injection of the nanodrug. Notably, with the Pt-nanodrug and Intralipid being metabolized in the liver, the Pt concentrations reach similar level at 72 hr, 10.1 ± 1.6 and 11.8 ± 3.7 μg/g wet weight (p > 0.1), without- and with-Intralipid pre-treatment, respectively. To increase and prolong the effectiveness of Intralipid, the administered dosages, time courses, and frequencies of Intralipid treatment need to be optimized for each chemotherapeutic regimen. Thus, we have designed a multi-dose protocol described in Fig. 1A, with two doses of DACHPt/HANP and six doses of Intralipid.

**Figure 2:**
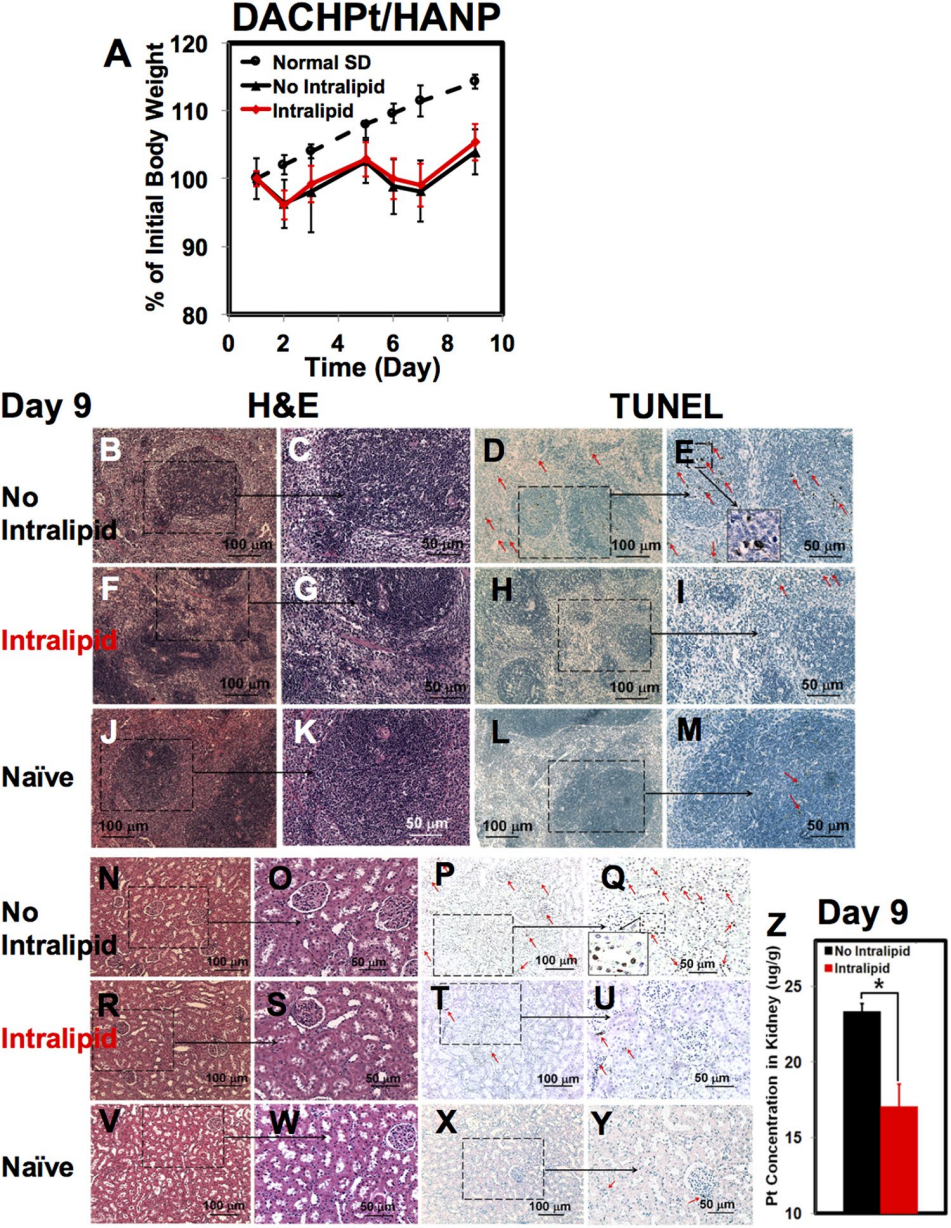
Intralipid treatment reduces the toxic side effects of DACHPt/HANP on day 9. (**A**) Changes of the body weight of rats upon administration of DACHPt/HANP, with and without treatment of Intralipid (N = 6). (**B**–**M**) Light microscopic images of H&E-stained (**B**,**C**,**F**,**G**, and **J**,**K**) and TUNEL-stained (**D**,**E**,**H**–**I** and **L**,**M**) spleen tissue sections. (**B**,**E**) are observed from the spleen-tissue sections of DACHPt/HANP administrated, but no Intralipid-treated, animals. (**F**–**I**) are from the Intralipid-treated animals. (**J**–**M**) are from the spleen-tissue sections of the naïve SD rats. (**C**,**E,G**,**I**,**K** and **M**) are the enlarged views of (**B**,**D**,**F**,**H**,**J** and **L**), respectively. Red arrows on (**D**,**E**,**I** and **M**) indicate the apoptotic cells. An enlarged view of the apoptotic spleen cells is shown as an example in (**E**). (**N**–**Y**) Light microscopic images of H&E-stained and TUNEL-stained kidney tissue sections. Red arrows on (**P**,**Q**,**T**,**U** and **Y**) indicate the apoptotic cells. An enlarged view of the apoptotic kidney cells is shown as an example in (**Q**). (**N**–**Q**) are observed from the kidney-tissue sections of DACHPt/HANP administrated, but no Intralipid-treated, animals. (**R**–**U**) are from the Intralipid-treated animals. (**V**–**Y**) are from the kidney-tissue sections of the naïve SD rats. (**Z**) Change of the Pt concentration in the kidney as measured by ICP-MS, upon Intralipid treatment, in the multiple-dose experiment.

Changes of the body weight caused by the administration of DACHPt/HANP and upon treatment with Intralipid 20% are shown in Fig. 2A (N = 6 for each group, i.e., Intralipid-treated and PBS-treated control groups). The first dose of DACHPt/HANP caused animal body weight to decrease ~4% in the 24-hr post administration, followed by slow recovery. The second dose of the Pt-containing nanodrug has caused body weight to decrease for 48 hr before slow recovery. There is no statistically significant difference between the Intralipid-treated group and the control group (no Intralipid treatment). The growth of the body weight of the naïve rats is shown for comparison (dash line in Fig. 2A).

Pathological studies [hematoxylin and eosin (H&E) staining and terminal deoxynucleotidyl transferase dUTP nick end labeling (TUNEL) assay] reveal that the second dose of DACHPt/HANP causes more damages to the spleen, kidney, and liver tissues (Figs 2B–E,N–Q, and S1B–E), comparing with a single dose of DACHPt/HANP as reported in our previous study^27^. Intralipid treatment significantly reduces the toxic side effects of multi-dose of DACHPt/HANP in the spleen (Fig. 2F–I). With DACHPt/HANP treatment, but no Intralipid, the spleen tissues are characterized by a disorder proliferation of mononuclear cells (Fig. 2B,C) and a large number of apoptotic cells (Fig. 2D,E). An enlarged view of apoptotic spleen cells is shown in Fig. 2E. Upon Intralipid treatment, the spleen cells show better morphology and distribution (Fig. 2F,G vs. B,C). Intralipid treatments significantly reduces the spleen cell apoptosis, especially at the germinal center of the white pulps (Fig. 2H,I vs. D,E). Very few apoptotic cells (red arrows in Fig. 2H,I) are observed in the Intralipid-treated group, comparable to the spleen tissues of the naïve rats (Fig. 2L,M).

The spleen weight of the animals is an indication of the toxic side effects of the nanodrug. As we reported previously^27^, a single dose of DACHPt/HANP can cause spleen enlargement and swelling. The ratio of the spleen weight/body weight for a naïve Sprague Dawley (SD) rat is 0.31 ± 0.06 (N = 3). Intralipid treatment alone does not change this ratio [0.28 ± 0.02 (N = 3)]. The ratio from a single-dose DACHPt/HANP-treated SD rat is 0.53 ± 0.08^27^. In our multi-dose experiments, we have found that two doses of DACHPt/HANP, without Intralipid, causes the spleen to shrink and reduces the ratio of spleen weight/body weight to 0.20 ± 0.03 (N = 6). With multi-doses of Intralipid treatment, this ratio is 0.26 ± 0.07 (N = 6). In Fig. S1A, the ratios are shown as the percentage of the normal level.

Nephrotoxicity is a major side effect of platinum drugs^32^ and our experimental nanodrug, DACHPt/HANP, is developed to reduce the nephrotoxicity^27^. Although multi-doses of DACHPt/HANP do not cause significant pathological changes in the kidney-tissue sections as shown by H&E staining (Fig. 2N,O), a few apoptotic cells are observed by the TUNEL staining (Fig. 2P,Q). An enlarged view of the apoptotic kidney cells is shown in Fig. 2Q. Intralipid treatment reduces the kidney damage caused by this Pt-containing nanodrug (Fig. 2R–U). The number of the apoptotic cells is significantly reduced at both glomerulus and tubules (Fig. 2T,U vs. P,Q). The kidney-tissue sections from the naïve SD rats (Fig. 2V–Y) are shown for comparison. We have found a 27% decrease of the Pt concentration in the kidney upon Intralipid treatment, as measured by inductively coupled plasma-mass spectrometry (ICP-MS) (Fig. 2Z). With DACHPt/HANP administration, but no Intralipid treatment, the Pt concentration in the kidney is 23.3 ± 2.6 (μg/g wet weight) on Day 9 when the animals were sacrificed. With Intralipid treatment, the Pt concentration decreases to 17.1 ± 0.1 (μg/g wet weight). We have also monitored the kidney function by the serum creatinine assay and observed the changes of the creatinine levels upon Intralipid treatment (Fig. S1N). However, from Day 5 to Day 9, we cannot detect significant changes in the serum creatinine levels upon Intralipid treatment.

The pathological and TUNEL analyses of the hepatic tissue sections are shown in Fig. S1B–M. A large amount of necrosis cells (black arrows on Fig. S1C, which is an enlarged view of Fig. S1B) and the apoptotic cells (red arrows on Fig. S1E, which is an enlarged view of Fig. S1D) are observed from the liver-tissue sections of the DACHPt/HANP-treated animals. The liver tissue sections from the naïve SD rats are shown for comparison (Fig. S1J–M). Probably because the liver damage caused by the second dosage of DACHPt/HANP was too severe, under the current experimental conditions, we have not observed significant improvements in the liver tissue upon Intralipid treatment. We have also monitored the changes in the liver function by the serum alanine aminotransferase (ALT) activity assay (Fig. S1O). From Day 5 to Day 9, we cannot detect significant changes in ALT levels upon the Intralipid treatment.

### Intralipid Reduces Toxic Side Effects of Abraxane

We have applied our “proof-of-concept” experimental design, as described in our previous study^27^, with some minor modifications, to test the effects of Intralipid 20% on the side effects of Abraxane. The treatment protocol is described in the Materials and Methods and shown in Fig. 1B. Intralipid 20% was administered to SD rats at the clinical dose (2 g/kg) using the clinical route (i.e., intravenously) 1 hr before i.v. injection of Abraxane (44 mg/kg, clinical dose for breast cancer treatment) and 24-hr post injection of the nanodrug. The tissue samples collected at 72-hr post injection were used for the histological analysis [N = 4 for Intralipid-treated and PBS-control group].

**Figure 3:**
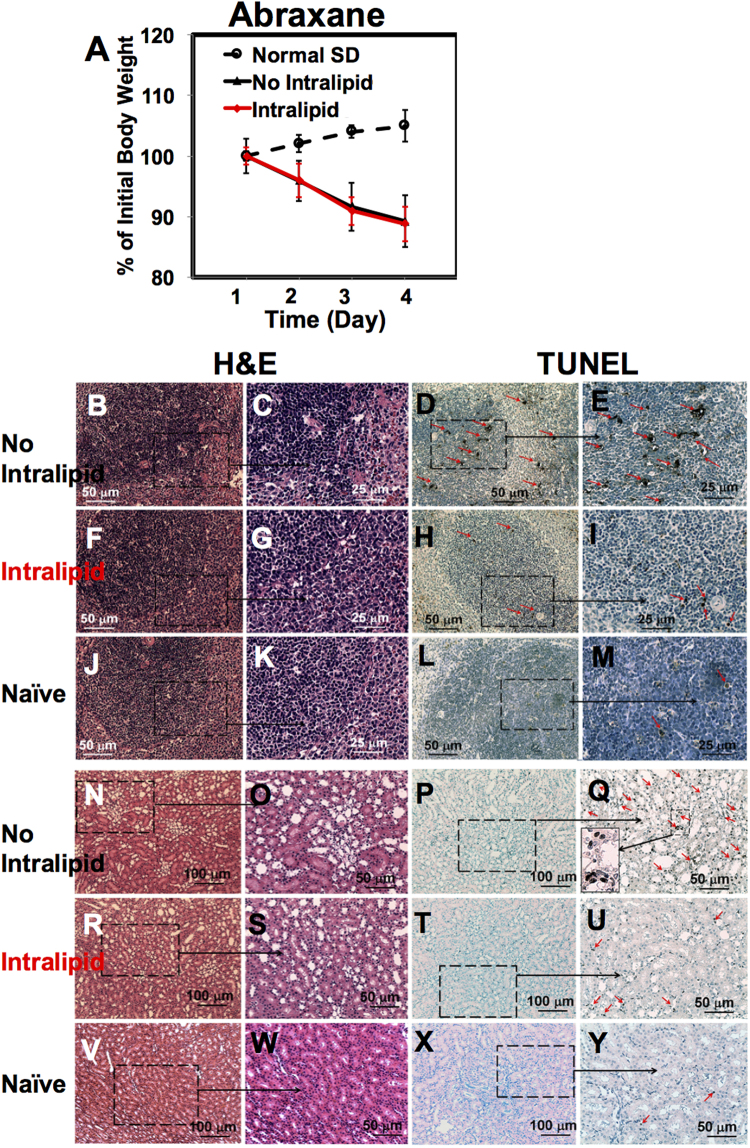
Intralipid treatment reduces the toxic side effects of Abraxane. (**A**) Changes of the body weight of rats upon administration of Abraxane, with and without treatment of Intralipid (N = 4). (**B**–**I**) Light microscopic images of the spleen tissue sections treated with Abraxane, with or without Intralipid. (**J**–**M**) are from the spleen-tissue sections of the naïve SD rats. Spleen tissue sections were stained with H&E (**B**,**C**,**F**,**G** and **J**,**K**) and TUNEL (**D**,**E**,**H**,**I**, and **L**,**M**). In (**D**,**E**,**H**,**I** and **M**), red arrows indicate the apoptotic cells. (**B**–**E**) are observed from the spleen-tissue sections of the Abraxane administrated, but no Intralipid-treated, animals. (**F**–**I**) are from the Intralipid-treated animals. (**N**–**Y**) Light microscopic images of the H&E-stained (**N**,**O**,**R**,**S**, and **V**,**W**) and the TUNEL-stained (**P**,**Q**,**T**,**U**, and **X**,**Y**) kidney tissue sections. The red arrows on (**Q**,**U** and **Y**) indicate the apoptotic cells. (**Q**) Shows an enlarged view of the apoptotic kidney cells.

Abraxane affects the animal body weight significantly, which indicates the toxicities of the drug. Animal body weight keeps decreasing by ~11% during the 72-hr post administration of Abraxane (Fig. 3A). At 72 hr, the body weight of the Abraxane-treated animals is ~18% lower than that of the naïve animals (dash line in Fig. 3A). Intralipid treatment could not change the loss of body weight significantly under current experimental conditions.

Fixed spleen tissue sections are analyzed with the H&E staining (Fig. 3B,C and F,G) and the TUNEL assay (Fig. 3D,E and H,I). With Abraxane administration, but no Intralipid, uneven distribution of mononuclear cells is observed from the H&E-stained spleen tissue (Fig. 3B,C). A large amount of apoptotic cells is observed at both white pulp and red pulp of the spleen tissue from the TUNEL assay (red arrows in Fig. 3D,E). Upon the Intralipid treatment, these damages are significantly reduced. The spleen cells are more evenly distributed in the Intralipid-treated group (Fig. 3F,G). Intralipid significantly reduces spleen cell apoptosis, especially at the white pulps (Fig. 3H,I). The spleen tissue sections from the naïve SD rats (Fig. 3J–M) are shown for comparison.

The decrease of the spleen weight caused by the administration of Abraxane, with or without treatment of Intralipid, are shown in Fig. S2A. The ratio of the spleen weight/body weight from Abraxane-treated SD rat is 0.19 ± 0.01 (N = 4). Intralipid treatment exhibits no significant change on this ratio (0.22 ± 0.03, N = 4). In Fig. S2A, the ratios are shown as the percentage of the normal level.

Intralipid treatment also reduces the toxic side effects of Abraxane in the kidney (Fig. 3N–Y). Although there is no significant pathological change from the H&E-stained kidney tissue (Fig. 3N,O), Abraxane treatment causes the kidney cell apoptosis, especially at the renal tubules, as indicated by red arrows on Fig. 3Q, which is an enlarged view of Fig. 3P. Intralipid administration significantly reduces the amount of apoptotic cells at the renal tubules (Fig. 3T,U vs. P,Q). Very few apoptotic cells are observed from the Intralipid-treated group (Fig. 3T,U), comparable to the kidney tissues of naïve rats (Fig. 3X,Y).

With respect to the liver, Abraxane treatment causes very mild hepatic cell necrosis (Fig. S2B,C) and apoptosis (Fig. S2D,E). There is no significant change upon the Intralipid treatment (Fig. S2F–I).

### Intralipid Reduces Toxic Side Effects of Marqibo

**Figure 4:**
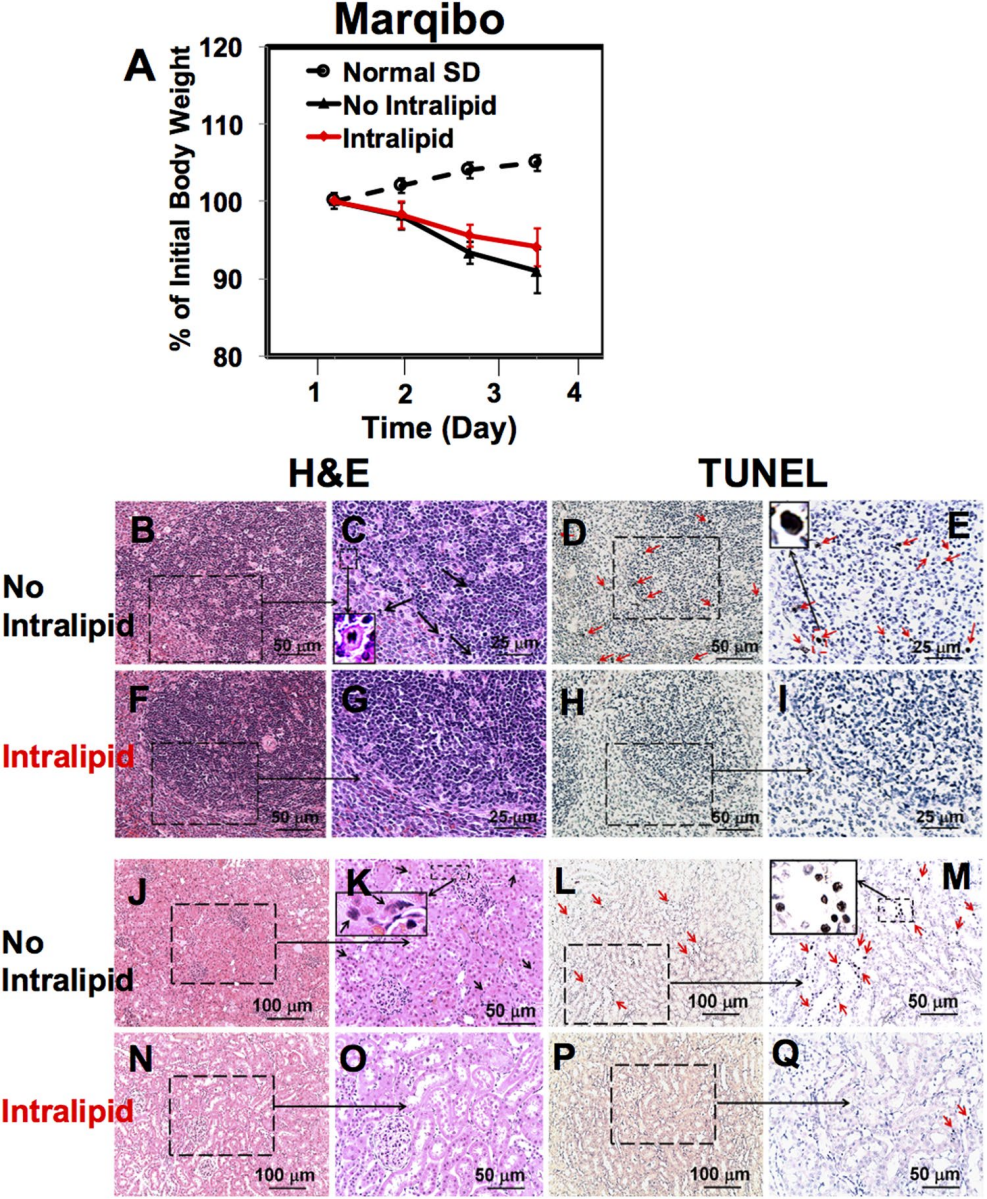
Intralipid treatment reduces the toxic side effects of Marqibo. (**A**) Changes of the body weight of the rats upon administration of Marqibo, with and without treatment of Intralipid (N = 6). (**B**–**I**) Light microscopic images of the spleen tissue sections treated with Marqibo, with or without Intralipid. The spleen tissue sections were stained with H&E (**B**,**C** and **F**,**G**) and TUNEL (**D**,**E** and **H**,**I**). In (**C**), the black arrows point to the mitotic cells and an enlarged view of the spleen mitotic cell is shown. In (**D**) and (**E**) The red arrows indicate the apoptotic cells. (**E**) Shows an enlarged view of the apoptotic cells. (**J**–**Q**) Light microscopic images of the (**H**,**E**)-stained (**J**,**K** and **N**,**O**) and the TUNEL-stained (**L**,**M** and **P**,**Q**) kidney tissue sections. In (**K**), the black arrows point to the mitotic cells and an enlarged view of kidney mitotic cell is shown. The red arrows on (**L**,**M** and **Q**) indicate the apopto tic cells. (**Q**) Shows an enlarged view of the apoptotic kidey cells.

Animal body weight reduces significantly when treated with Marqibo (Fig. 4A, N = 6), indicating the toxic side effects of this nanodrug. We have observed a large number of mitotic cells in the liver (black arrows in Fig. S3B,C), and a few in the spleen and the kidney (black arrows in Fig. 4B,C and J,K). In Figs 4C,K and S3C, an enlarged view of the mitotic cell is shown as an example. Intralipid treatment significantly reduces the amount of mitotic cells in the spleen and the kidney for Marqibo administration (Fig. 4F,G and N,O). With Intralipid treatment, the mononuclear cells in the spleen show more uniformly distribution (Fig. 4F,G vs B,C). The size of the spleen is not affected by Marqibo significantly (Fig. S3A). Intralipid appears to reduce the number of mitotic cells in the liver as well, although there is still quite an amount of the mitotic cells in the liver tissue (Fig. S3F,G). The spleen, kidney, and liver tissue sections from the naïve rats are shown in (Figs 3J–M,V–Y and S1J–M) for comparison.

The large amount of the mitotic cells might be explained by the mechanism of Vincristine, which binds tubulin and causes the microtubule depolymerization, metaphase arrest, and apoptotic death of cells undergoing mitosis^33^. Although Abraxane is also a mitotic inhibitor, we do not observe this large amount mitoses in the tissues when treated with Abraxane (Figs 3B,C,N,O and S2B,C).

We have observed a large amount to apoptotic cells in the spleen and the kidney when treated with Marqibo (red arrows in Fig. 4D,E and L,M). Intralipid greatly reduces the amount of the apoptotic cells in the spleen and kidney (Fig. 4H,I and P,Q). Consistent with the reduction of the mitotic and the apoptotic cells in the kidney, serum creatinine assay also shows the protective effects of Intralipid (Fig. S3J). Very few apoptotic cells are observed in the liver tissue sections (Fig. S3D,E) and we have not detected the change of serum ALT activity when treated with Marqibo (Fig. S3K).

### Intralipid Reduces Toxic Side Effects of Onivyde

Onivyde is a prodrug^23^. After we have observed the beneficial effects of Intralipid for the above three anti-cancer nanodrugs, we decide to test whether Intralipid can reduce the toxic side effects of Onivyde.

**Figure 5:**
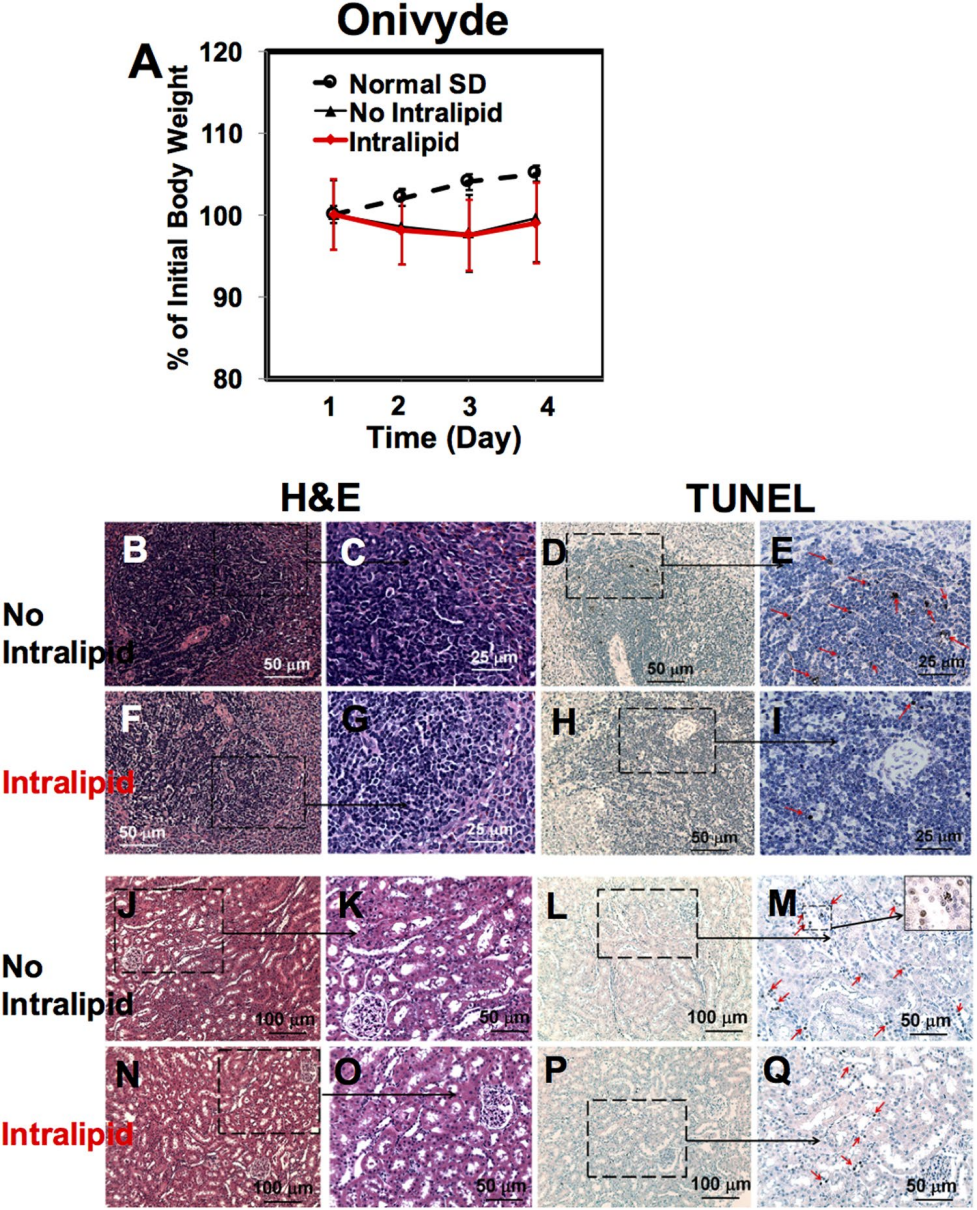
Intralipid treatment reduces the toxic side effects of Onivyde. (**A**) Changes of the body weight of rats upon administration of Onivyde, with and without treatment of Intralipid (N = 6). (**B**–**I**) Light microscopic images of the spleen tissue sections treated with Onivyde, with or without Intralipid. The spleen tissue sections were stained with H&E (**B**,**C** and **F**,**G**) and TUNEL (**D**,**E** and **H**,**I**). In (**E**) and (**I**), the red arrows indicate the spleen apoptotic cells. (**J**–**Q**) The light microscopic images of the H&E-stained (**J**,**K** and **N**,**O**) and the TUNEL-stained (**L**,**M** and **P**,**Q**) kidney tissue sections. The red arrows on (**M** and **Q**) indicate the kidney apoptotic cells.

Animals show a mild loss of the body weight post treatment of Onivyde (Fig. 5A, N = 6). Intralipid treatment has no significant effects on the body weight. Consistent with a mild loss of the body weight, Onivyde shows mild toxicities in the spleen, kidney, and liver (Figs 5B–E and J–M, and S4B,E). The H&E-stained spleen-tissue sections from Onivyde administrated, without and with Intralipid treatment (Fig. 5B,C and F,G), look similar to the spleen tissue of the naïve rats (Fig. 3J,K). Onivyde treatment causes mild spleen cell apoptosis as shown by the red arrows in Fig. 5E, which is an enlarged view of Fig. 5D. Intralipid treatment reduces the amount of the apoptotic cells (Fig. 5H,I). Onivyde treatment also slightly reduces the size of the spleen (Fig. S4A). The ratio of the spleen weight/body weight from Onivyde-treated SD rat is 0.21 ± 0.02 (N = 6). Intralipid treatment exhibits no change in this ratio (0.21 ± 0.01, N = 6).

There is no significant change in the H&E-stained kidney-tissue sections from the rats treated with Onivyde, without or with Intralipid treatment, compared with those of the naïve rats (Figs 5J,K,N,O vs. 2V,W). A few apoptotic cells are observed at the renal tubules when treated with Onivyde (Fig. 5L,M). Intralipid treatment significantly reduces the number of the apoptotic cells at the renal tubules (Fig. 5P,Q). Serum creatinine level assay cannot detect this change (Fig. S4N). With regard to the liver-tissue sections, very few necrosis cells (Fig. S4B,C) and apoptotic cells (Fig. S4D,E) are observed from the Onivyde-treated group. Intralipid treatment has no harmful effect to the liver tissue (Fig. S4F–I). We have verified this result by testing the liver function using blood serum ALT activity assay (Fig. S4O). There is no significant change in the serum ALT activity from the animals treated with Onivyde with or without Intralipid.

### Effects of Intralipid Treatment on the Anti-tumor Efficacy of DACHPt/HANP in a HT-29 Xenograft Mouse Model

**Figure 6:**
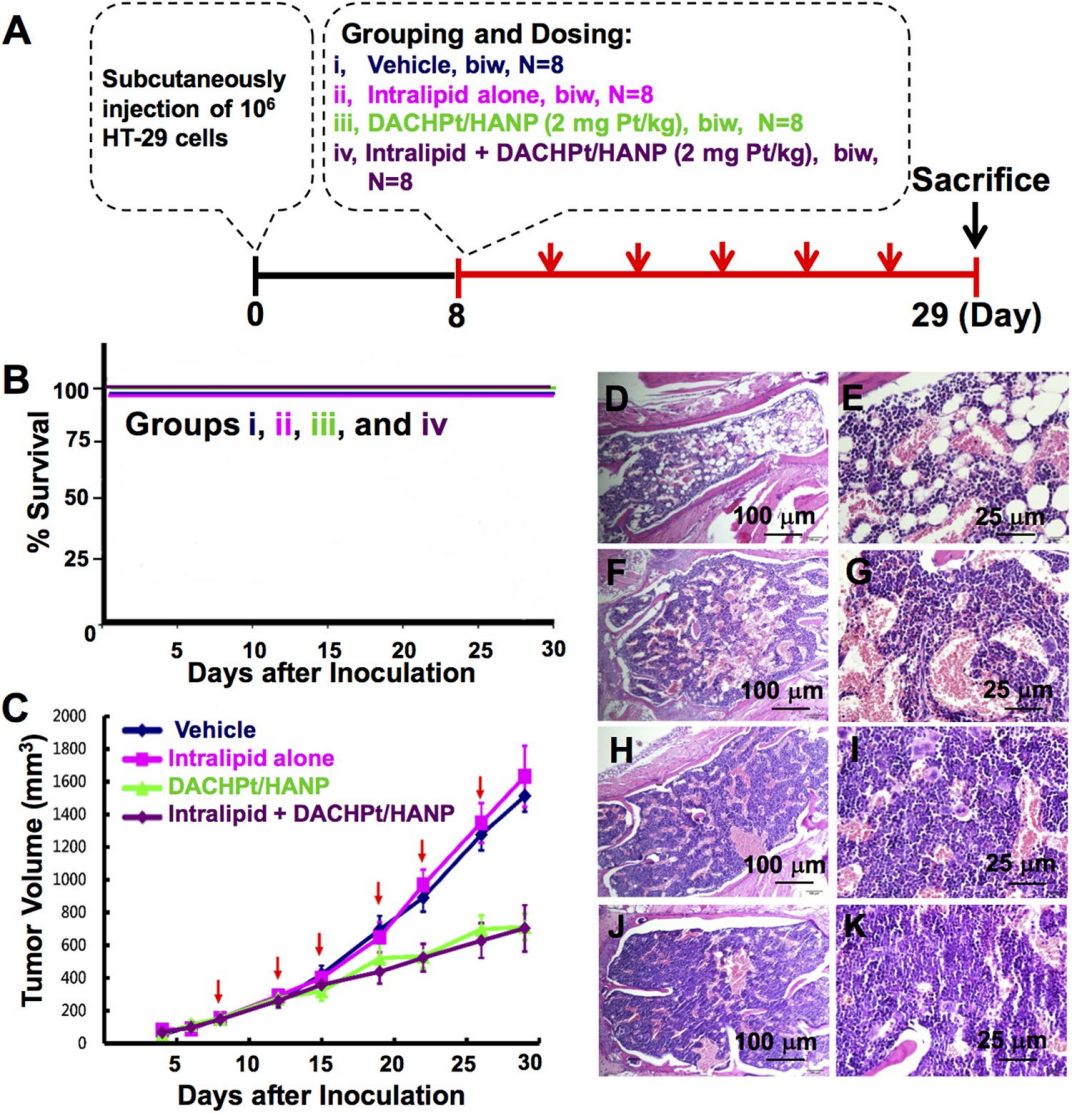
Effects of Intralipid treatment on the tumor growth and the anti-tumor efficacy of DACHPt/HANP as well as the survival rate in a HT-29 xenograft tumor model. (**A**) Treatment protocol. (**B**) Survival rate of HT-29 bearing BALB/c nude mice for the four treatment groups. (**C**) Changes of the tumor size upon treatment with vehicle, Intralipid alone, and DACHPt/HANP at 2 mg Pt/kg with or without Intralipid administration. H&E staining of the bone marrow is shown in (**D**–**K**). (**D**,**E**) DACHPt/HANP treatment, with no Intralipid, causes moderate/severe depletion of erythroid and myeloid precursor cells in the bone marrow. (**F**,**G**) Intralipid treatment significantly reduces this damage. (**H**,**I**) and (**J**,**K**) Bone marrow from (i) vehicle-treated, and (ii) Intralipid alone group.

We have conducted a study to show the effect of Intralipid on the anti-cancer efficacy and survival rate using an experimental anti-cancer nanodrug, DACHPt/HANP, with a HT-29 human colon cancer xenograft mouse model. When the tumor volume reached sizes of 100 to 200 mm^3^, mice were randomly divided into four treatment groups (Fig. 6A): (i) vehicle; (ii) Intralipid alone; and (iii-iv) DACHPt/HANP (2 mg Pt/kg), without and with Intralipid 1-hr pre-treatment. Intralipid treatment shows no negative effect on the animal body weight (Fig. S5). As shown in Fig. 6B, the animal survival rate is 100% for groups (i–iv). Thus, Intralipid treatment exhibits no harmful effect for the survival rate.

The changes of the tumor volume for the groups: (i) vehicle; (ii) Intralipid alone; (iii–iv) DACHPt/HANP (2 mg Pt/kg), without and with Intralipid 1-hr pre-treatment, are shown in Fig. 6C. First of all, there is no significant difference in the tumor volume when we compare the vehicle-treated and the Intralipid-alone group. Intralipid does not promote tumor growth and is a safe agent for the mice tested to reduce toxic side effects of the drug. Second, when we compare the treatment groups (iii) DACHPt/HANP and (iv) the Intralipid pre-treatment before injecting DACHPt/HANP, there is no significant difference in the tumor volume. Thus, Intralipid treatment does not reduce the anti-tumor efficacy of the nanodrug.

In this experiment, we have also found that Intralipid treatment can reduce the toxicities in the bone marrow, which is also an important organ of the RES, caused by the Pt-nanodrug (Fig. 6D–G). The degree of lesions in bone marrow is graded from one to five depending on severity^34^: Grade 1 = minimal (<1%); Grade 2 = slight (1–25%); Grade 3 = moderate (26–50%); Grade 4 = moderate/severe (51–75%); Grade 5 = severe (76–100%). DACHPt/HANP causes severe damage, i.e., Grade 4, to the bone marrow of the mice, exhibiting moderate/severe depletion of erythroid and myeloid precursor cells (Fig. 6D,E). Upon Intralipid treatment, these damages are greatly reduced and the severity is graded as Grade 2~3, i.e., slight to moderate, (Fig. 6F,G). H&E-stained bone marrow from the vehicle-treated and the Intralipid alone group are shown for comparison (Fig. 6H–K).

## Discussion

Nanodrugs are making significant impacts on the treatment of many human diseases, e.g., cancer^1^, autoimmune diseases^35^, inflammation^36^, regenerative medicine^37^, fungal infections^38^, vaccines^39^, anesthetics^40^, and macular degeneration^41^. RES uptake is important for the pharmacokinetics (PK), pharmacodynamics (PD), efficacy of drug delivery, as well as for the off-target toxicities of the nanodrugs. A large volume of work has been conducted to reduce the RES uptake of nanodrugs in the hope to reduce their toxicity and to increase their bioavailability. A major accomplishment in this field is to make “stealth” nanoparticles, by coating the particle surface with hydrophilic polymers/surfactants, and/or formulation with biodegradable copolymers with hydrophilic segments, such as polyethylene glycol (PEG), dextrane, poloxamine, and poloxamer^14,42^. PEG is the most commonly used non-ionic hydrophilic polymer to make “stealth” nanoparticles. The first approved PEGylated product, Doxil/Caelyx, has already been in the clinic for about 20 years^43,44^. Clinical experience and laboratory research of this polymer have shown not only the benefits, but also its unfavorable effects^45^, such as the hypersensitivity reactions induced by the polymer itself or by its side products formed during synthesis^46,47^, the unexpected changes in the pharmacokinetic behavior caused by the accelerated blood clearance (ABC) phenomenon^48–50^, and the toxic side products and an antagonism arising from the degradation under stress or upon exposure to oxygen^45^.

Our Intralipid methodology could also be a valuable addition/supplement to the “stealth” strategies^26–28^. Intralipid was approved in the United States in 1972 and its safety has been shown in clinical practice^29^. Intralipid 20% is composed of 20% soybean oil, 1.2% egg-yolk phospholipids, and 2.25% glycerol^29^. The major fatty acid constituents are linoleic acid (44–62%), oleic acid (19–30%), palmitic acid (7–14%), linolenic acid (4–11%), and stealic acide (1.4–5.5%). We have conducted “proof-of-concept” studies to show that the Intralipid treatment can change the PK/PD profile of nanoparticles and toxicological profile of an anti-cancer nanodrug^26–28^. Here, we have extended our study to show that Intralipid treatment can improve the delivery for four different types of anti-cancer nanodrugs, an experimental DACHPt/HANP and three FDA approved nanodrugs Abraxane, Marqibo, and Onivyde, using a rat model. The anti-cancer mechanisms of these four drugs are quite different. DACHPt/HANP induces cancer cell apoptosis by causing cross-linking of DNA and DNA-protein^51^. Paclitaxel targets tubulin and blocks the progression of mitosis, through the activation of the mitotic checkpoint (also known as the spindle assembly checkpoint)^52^. Vincristine also binds tubulin, but causes microtubule depolymerization, metaphase arrest, and apoptotic death of cells undergoing mitosis^33^. Irinotecan is a prodrug, thus it must be metabolized in the liver and intestine to form its active metabolite SN-38, a Topoisomerase I inhibitor^23,53^. Intralipid methodology does improve the delivery of all these four nanodrugs by reducing their toxicities in the RES and kidney.

Intralipid is metabolized in the liver actively as reported that the circulating ketone bodies increased ~100% in 30 min after i.v. administration of Intralipid^30^. The blood half-life of Intralipid in rats is 8.7 ± 3.0 min (by i.v. bolus administration)^30,31^. Thus, multi-doses of Intralipid may be necessary for clinical applications of nanodrugs. Our positive findings suggest that our Intralipid methodology could become a new way to deliver anti-cancer and other nanodrugs and will encourage additional animal experiments and clinical studies to design the optimal way of using Intralipid to deliver the anti-cancer and other nanodrugs.

## Concluding Remarks

The results described here clearly show that in rodents, Intralipid methodology can reduce the off-target toxicities of the four anti-cancer nanodrugs tested in RES and kidney and can also increase the bioavailability of the Pt-nanodrug^27^. We believe that our methodology can have important implications for the delivery of nanodrugs in clinical settings and warrants additional studies to optimize the Intralipid methodology in relation to these drugs. A critical limitation in the current delivery of the nanodrugs to patients is the amount of these powerful drugs that a patient can tolerate. Using the Intralipid methodology, a physician could have two options. Since Intralipid can reduce the off-target toxicities in liver, spleen, and kidney, a physician could increase the dosage of a nanodrug to kill as many cancer cells as possible. If the Intralipid treatment can improve the bioavailability of the drug as shown in DACHPt/HANP, thus can improve the delivery of the drug, a physician could reduce the dosage of the drug without affecting the efficacy of the drug. In both cases, it is a win-win situation, namely to improve the quality of life of a patient due to a reduction of the off-target side effects of a nanodrug and to reduce the dosage of the expensive nanodrugs used in chemotherapy. A unique feature of our methodology is that we do not need to make any modifications on the existing FDA approved nanodrugs and nanocarriers, thus facilitating the clinical translation of our methodology.

## Materials and Methods

### Materials and Animals

Intralipid 20% was purchased from Fresenius Kabi (Bad Homburg, Germany). DACHPt/HANP was synthesized and characterized as described previously^27^. Abraxane was purchased from Celgene Corporation (Summit, NJ). Onivyde was purchased from Merrimack Pharmaceuticals, Inc (Cambridge, MA). Marqibo was purchased from Talon Therapeutics, Inc (Foster City, CA). Phosphate-buffered-saline (PBS) was purchased from Mediatech (Manassas, VA). 0.9% Sodium chloride injection UPS was purchased from Baxter Healthcare Corporation (Deerfield, IL).

Male SD rats with an indwelling jugular vein catheter implanted were purchased from Harlan Laboratories (Indianapolis, IN). All experiments involving animal subjects were approved by the Institutional Animal Care and Use Committee of the Carnegie Mellon University and the Industrial Technology Research Institute (ITRI) of Taiwan. Animal care was provided in accordance with the Guide for the Care and Use of Laboratory Animals, published by the NIH (Eighth Edition, 2011) and the guidance of the Associated for Assessment and Accreditation of Laboratory Animal Care (AAALAC).

### Preparation of nanodrugs

DACHPt/HANP was reconstituted with sterilized deionized water and used within 2 hr after reconstitution^27^. Abraxane was reconstituted with 0.9% sodium chloride and used within 2 hr after reconstitution according to the Instruction for Preparation. Marqibo was prepared according to the Pharmacy Instructions for Preparation (http://www.marqibo.com/pdf/Marqibo-Preparation-Mix-Brochure.pdf) and used within 4 hr after reconstitution. Onivyde was diluted with appropriate volume of 0.9% sodium chloride according to the Instruction for Preparation and used within 4 hr after dilution.

## Experimental Design

### Experimental Design for Using Multiple Doses of Intralipid to Investigate the Effect on Delivery of Two Doses of DACHPt/HANP

Male Sprague Dawley (SD) rats (body weights between 260 and 280 g) with an indwelling jugular vein catheter implanted were used. Experimental procedures are shown in Fig. 1A. Rats (N = 6 for each group, i.e., the Intralipid-treated group and the control group) were treated with DACHPt/HANP (2 mg Pt/kg) intravenously twice a week (on Days 1 and 5). On Days 1 and 5, Intralipid 20% was intravenously administered (clinical route) at a dosage of 2 g/kg 1 hr before the administration of the nanodrug. On Days 2, 3, 6, and 7, the rats were administered 2 g/kg of Intralipid 20% or PBS (as the control) each day. The body weight of the animals was recorded every day. Blood was drawn, through the implanted indwelling jugular vein catheter, everyday or every other day. Blood serum was prepared for serum alanine aminotransferase (ALT) activity assay for the liver function and serum creatinine assay for the kidney function. The animals were sacrificed on Day 9. The weights of liver, spleen, and kidney were recorded. Tissue samples were fixed in 4% paraformaldehyde (PFA) for histological analyses.

### Experimental Design for Using Two Doses of Intralipid to Investigate the Effect on the Delivery of FDA Approved Anti-cancer Nanodrugs, Abraxane, Marqibo, and Onivyde

Experimental procedures are shown in Fig. 1B. Intralipid 20% was administered intravenously at 2 g/kg one hr before i.v. treatment (clinical route) of nanodrugs (clinical dose: Abraxane 44 mg/kg; Marqibo 0.38 mg/kg; and Onivyde 11.86 mg/kg). 24-hr post nanodrug treatment, the second dose of Intralipid (2 g/kg) was administered to the animals. PBS was administered to the control animals. The animals were sacrificed at 72 hr. Tissue samples (liver, spleen, and kidney) were weighed and fixed in 4% PFA for histological analyses. N = 4 for Abraxane experiment and N = 6 for Marqibo and Onivyde experiments, for each group, i.e., the Intralipid-treated and the control group.

### Pt Levels in Tissues

The tissue samples were prepared and analyzed for Pt concentration by inductively coupled plasma-mass spectrometry (ICP-MS) [NexION 300X (PerkinElmer, Waltham, MA)] as described in our previous study^27^. Briefly, the wet weight of each tissue sample was recorded. About 0.5 mL of tissue homogenate was digested in HNO_3_ (1 mL) at 60 °C overnight. The HNO_3_-digested samples were evaporated and then re-dissolved in 2 N HCl (0.5 mL). Suitable dilutions were prepared using 5% HCl to reach a final Pt concentration in the range of 0.02 to 1 part per million (ppm). ^194^Pt, ^195^Pt, and ^196^Pt isotopes were analyzed and similar results were obtained from the measurement of these three isotopes. The Pt concentrations shown in this manuscript were calculated from the measurements of ^194^Pt concentration^27^.

### ALT Activity Assay and Creatinine Colorimetric Assay

The activity of ALT in serum was measured by using the ALT Activity Assay Kit purchased from Sigma-Aldrich, according to the supplier’s protocol. Serum creatinine level was measured by using the Creatinine Colorimetric/Fluorometric Assay Kit purchased from BioVision.

### HT-29 Xenograft Mouse Model

Human colon cancer cell line, HT-29 was cultured in McCoy’s 5 A medium supplemented with 10% fetal bovine serum (FBS) and maintained in a humidified incubator at 37 °C with 5% CO_2_. Female BALB/c nude mice (CAnN.Cg-*Foxn1* ^*nu*^/CrlBltw) at 6 to 8 weeks were purchased from BioLASCO Taiwan Co., Ltd (Ilan, Taiwan).

In brief, HT-29 cells (1 × 10^6^) were suspended in PBS with 25% Matrigel (BD Biosciences, Bedford, MA) and subcutaneously implanted into the right flank of the mouse. The administration of Intralipid and DACHPt/HANP was initiated when the mean of tumor volume reached 100 to 200 mm^3^ (8 days after HT-29 cells inoculation). Mice were divided to four groups, including: (i) vehicle; (ii) Intralipid alone; and (iii and iv) DACHPt/HANP at 2 mg Pt/kg with or without Intralipid pre-treatment. N = 8 for each group. DACHPt/HANP was intravenously injected in tail vein of mice twice a week (biw) for three weeks in 10 mL/kg per mouse. All treatments of Intralipid were at dose of 2 g/kg. Intralipid was intravenously administered 1 hr before each intravenous injection of DACHPt/HANP in the Intralipid pre-treatment group. Tumor volume was monitored twice a week. Tumor size was measured by Caliper and converted into tumor volume (mm^3^, V) using the formula: V = 0.5 × [length × (width)^2^]. After three weeks of treatment, mice were sacrificed and bone marrow specimens were fixed in 10% normal formalin for H&E staining.

### Pathological Analysis and TUNEL Assay

Histological examinations and TUNEL assays for spleen, liver and kidney tissue sections of rats were performed by the Transplantation Pathology Laboratory and the Neuropathology Laboratory of the Department of Pathology of University of Pittsburgh School of Medicine (Pittsburgh, PA). Paraffin-embedded 5-μm sections were stained with H&E, or were used for the TUNEL staining. For histopathological diagnosis, slides were examined by light microscopy and photomicrographs were taken using a Moticam 2300 camera mounted on an Olympus Provis microscope with Mtic Images Plus 2.0 software. H&E staining and examination of bone marrow specimens of mice were carried out by the Animal Pathology Laboratory of ITRI.

### Statistical Analysis

Survival analysis was done according to the Kaplan-Meier method with GraphPad Prism 6.01 software. Statistical analysis was carried out with the Student’s *t* test. A *p* value < 0.05 was considered statistically significant.

## Electronic supplementary material

Supplementary Information
