# Supplementary material for: A New Approach to Deliver Anti-cancer Nanodrugs with Reduced Off-target Toxicities and Improved Efficiency by Temporarily Blunting the Reticuloendothelial System with Intralipid
Source: Sci Rep. 2017 Nov 23;7:16106. doi: 10.1038/s41598-017-16293-6 (PMC5701028; doi:10.1038/s41598-017-16293-6)
Supplement: Supplementary file 1 — Supplementary Information [file 41598_2017_16293_MOESM1_ESM.pdf]

Supplementary Information

A New Approach to Deliver Anti-cancer Nanodrugs with Reduced Off-target Toxicities and Improved Efficiency by Temporarily Blunting the Reticuloendothelial System with Intralipid

Li Liu[1,2], Qing Ye[1,3], Maggie Lu[4], Shih-Ta Chen[4], Hsiang-Wen Tseng[4], Ya-Chin Lo[4], and Chien Ho[1*]

[1] Department of Biological Sciences, Carnegie Mellon University, Pittsburgh, PA

[2] Present address: National Institute of Neurological Disorders and Stroke, National Institute of Health, Bethesda, MD

[3] Present address: Department of Neurology, University of Pittsburgh School of Medicine, Pittsburgh, PA

[4] Drug Delivery Technology Department, Targeted Drug and Delivery Technology Division, Biomedical Technology and Device Research Laboratories, Industrial Technology Research Institute, Hsinchu, Taiwan

[*] Correspondence to Chien Ho [chienho@andrew.cmu.edu], Department of Biological Sciences, Carnegie Mellon University, 4400 Fifth Ave, Pittsburgh, PA 15213, USA

Supplementary Figure Captions

Fig S1. Changes of (**A**) the spleen weight, (**B-M**) liver pathological features, (**N**) serum creatinine level, and (**O**) ALT activity of rats upon administration of DACHPt/HANP (2 mg Pt/kg), with and without Intralipid treatment. N=6 for each group, i.e., the Intralipid-treated group and the control group. In (**A**), the spleen weight/body weight ratios are shown as the percentage of the normal level. (**B-C, F-G,** and **J-K**) are the H&E-stained sections of the liver tissue; (**D-E, H-I,** and **L-M**) are the TUNEL-stained sections of the liver tissue. (**B-E**) are from the tissue sections of the DACHPt/HANP administered, PBS-treated control group; (**F-I**) are from the Intralipid-treated group. (**J-M**) are the tissue sections of the naïve SD rats. In **E** and **I**, the red arrows indicate the apoptotic cells and an enlarged view of the apoptotic cell is shown. In **N** and **O**, $*p<0.05$.

Fig S2. Changes of (**A**) the spleen weight, and (**B-M**) liver pathological features of rats upon administration of Abraxane, with and without treatment with Intralipid. N=4 for each group, i.e., the Intralipid-treated group and the PBS-treated control group. In (**A**), the spleen weight/body weight ratios are shown as the percentage of the normal level. (**B-C, F-G,** and **J-K**) are from the H&E-stained tissue sections; (**D-E, H-I,** and **L-M**) are from the TUNEL-stained tissue sections. (**B-E**) are from the tissue sections of the Abraxane administered, PBS-treated control group; (**F-I**) are from the Intralipid-treated group, (**J-M**) are from the tissue sections of the naïve SD rats. In **E** and **I**, the red arrows point to apoptotic cells and an enlarged view of the apoptotic cell is shown.

Fig S3. Changes of (**A**) the spleen weight, (**B-I**) the liver pathological features, (**J**) the serum creatinine level, and (**K**) the ALT activity of rats upon administration of Marqibo, with and without Intralipid treatment. N=6 for each group, i.e., the Intralipid-treated group and the PBS-treated control group. (**B-C** and **F-G**) are from the H&E-stained liver tissue sections; (**D-E** and **H-I**) are from the TUNEL-stained liver tissue sections. (**B-E**) are from the tissue sections of the Marqibo administered, PBS treated control group; (**F-I**) are from the Intralipid-treated group. In **C** and **G**, the black arrows point to the mitotic cells and an enlarged view of the mitotic cell is shown. In **E** and **I**, the red arrows point to the apoptotic cells. In **J**, $^{*}p<0.05$.

Fig S4. Changes of (**A**) the spleen weight, (**B-M**) the liver pathological features, (**N**) the serum creatinine level, and (**O**) the ALT activity of rats upon administration of Onivyde, with and without treatment with Intralipid. N=6 for each group, i.e., the Intralipid-treated group and the control group. In (**A**), the spleen weight/body weight ratios are shown as the percentage of the normal level. (**B-C, F-G,** and **J-K**) are from the H&E-stained liver tissue sections; (**D-E, H-I,** and **L-M**) are from the TUNEL-stained liver tissue sections. (**B-E**) are from the tissue sections of the Onivyde administered, PBS-treated control group; (**F-I**) are from the Intralipid-treated group. (**J-M**) are from the tissue sections of the naïve SD rats. In **E, J,** and **M**, the red arrows point to the apoptotic cells.

Fig S5. Changes of the body weight of the BALB/c nude mice bearing human colon cancer HT-29 xenografts upon treatment (as indicated by the red arrows) with vehicle,

Intralipid alone, and DACHPt/HANP at 2 mg Pt/kg with or without Intralipid pre-treatment.

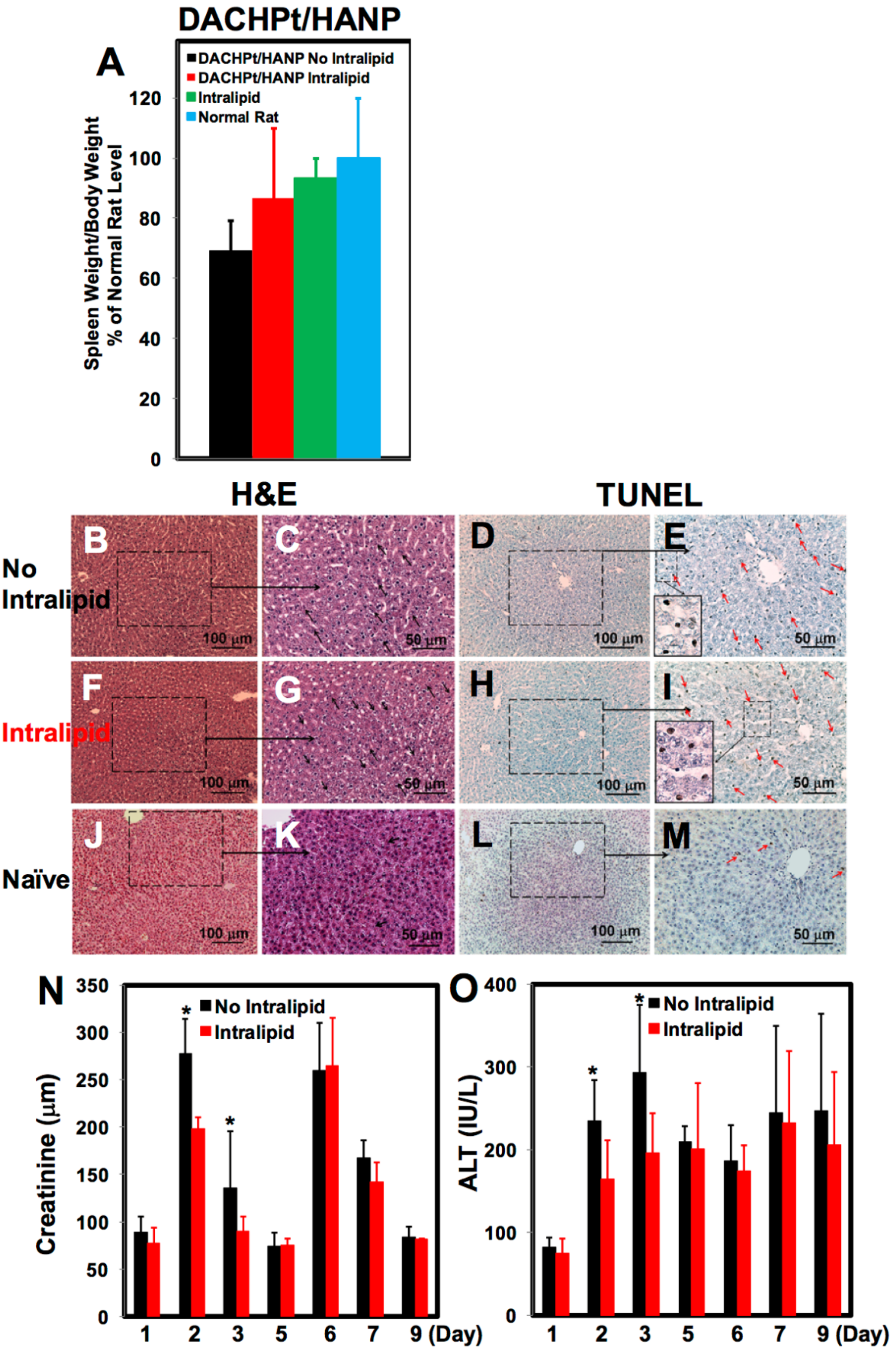
DACHPt/HANP
A
DACHPt/HANP No Intralipid
DACHPt/HANP Intralipid
Intralipid
Normal Rat
Spleen Weight/Body Weight
% of Normal Rat Level
120
100
80
60
40
20
0
H&E
TUNEL
B
C
D
E
No
Intralipid
100 μm
50 μm
F
G
H
I
Intralipid
J
K
L
M
Naïve
N
No Intralipid
Intralipid
Creatinine (μm)
350
300
250
200
150
100
50
1 2 3 5 6 7 9 (Day)
O
ALT (IU/L)
400
1 2 3 5 6 7 9 (Day)

Fig S1.

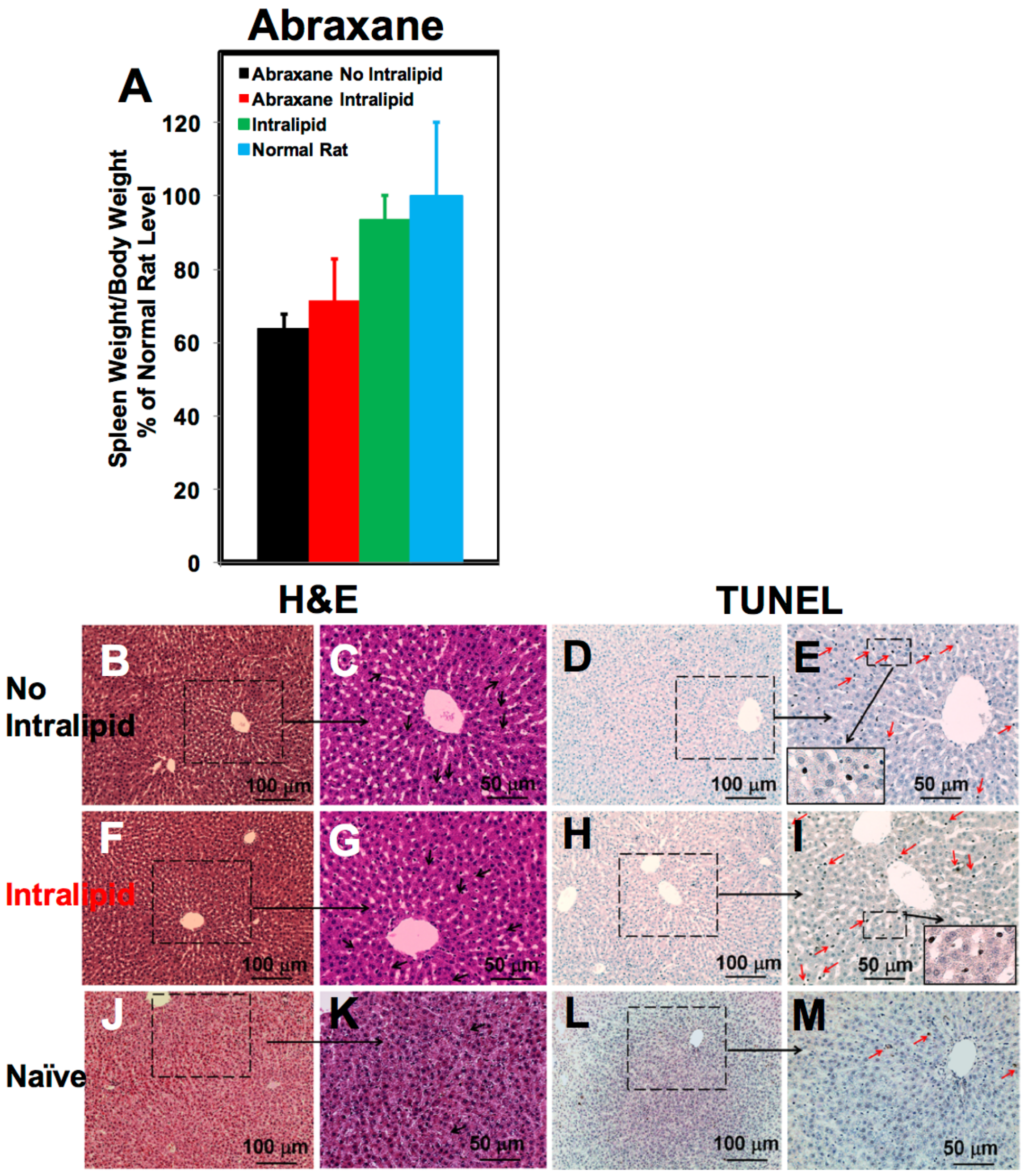
Abraxane
A
Abraxane No Intralipid
Abraxane Intralipid
Intralipid
Normal Rat
Spleen Weight/Body Weight
% of Normal Rat Level
120
100
80
60
40
20
0
H&E
TUNEL
No Intralipid
Intralipid
Naïve
B
C
D
E
F
G
H
I
J
K
L
M
100 µm
50 µm

Fig S2.

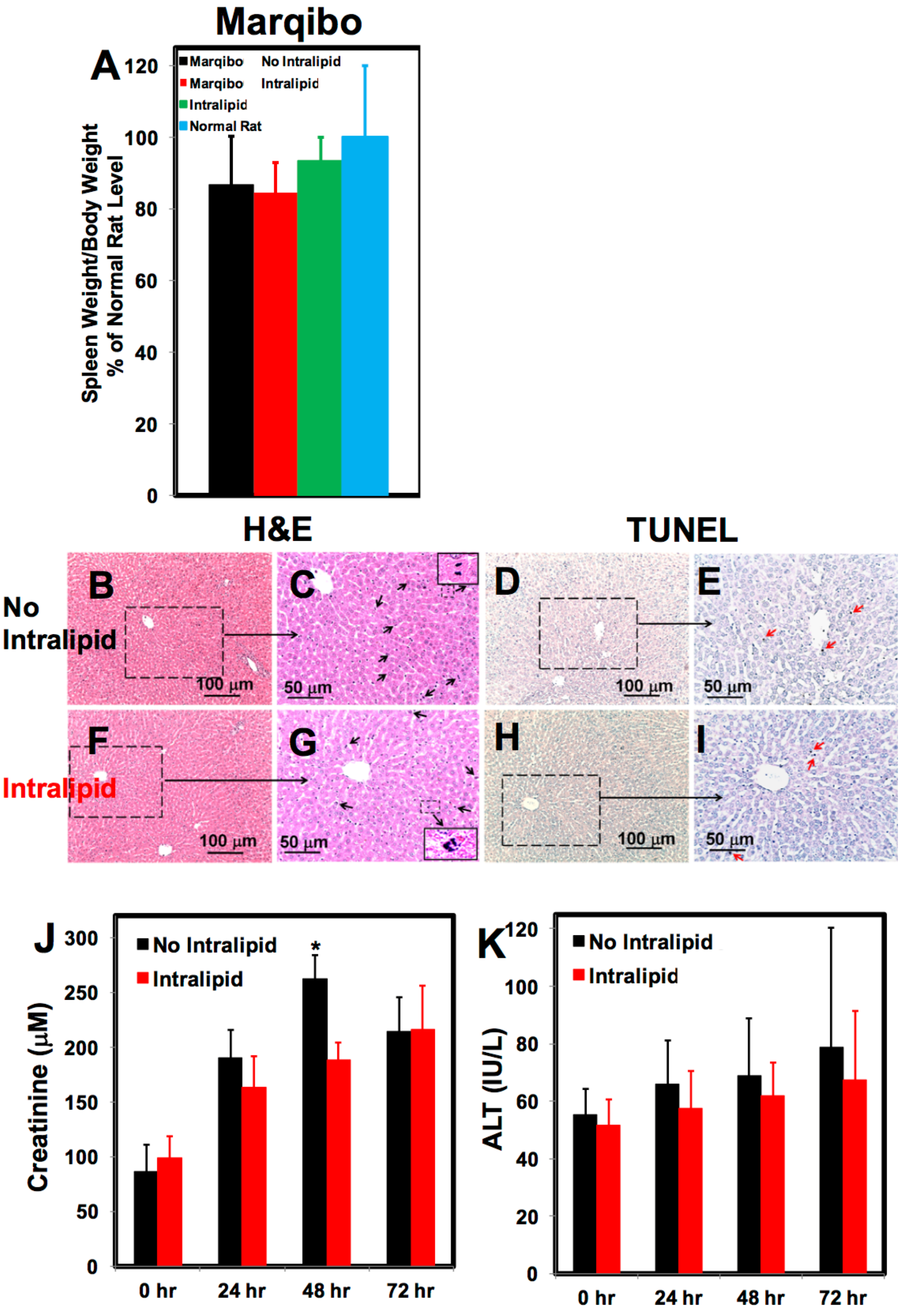
Marqibo
A
Marqibo No Intralipid
Marqibo Intralipid
Intralipid
Normal Rat
Spleen Weight/Body Weight
% of Normal Rat Level
120
100
80
60
40
20
0
H&E
TUNEL
No
Intralipid
Intralipid
B
C
D
E
F
G
H
I
100 µm
50 µm
J
No Intralipid
Intralipid
Creatinine (µM)
300
250
200
150
100
50
0
*
0 hr
24 hr
48 hr
72 hr
K
ALT (IU/L)
120
100
80
60
40
20
0

Fig S3.

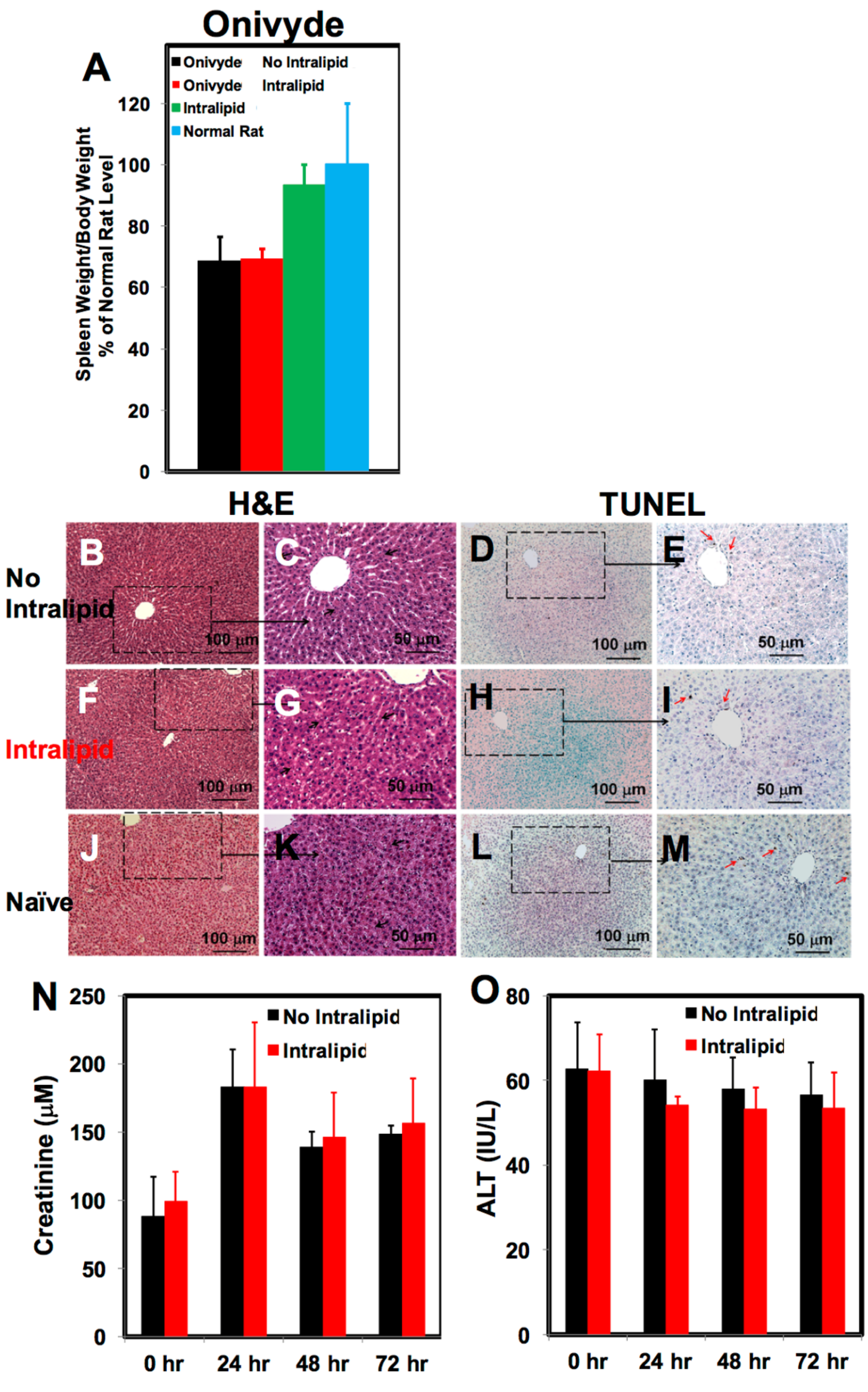
Onivyde
A
Onivyde No Intralipid
Onivyde Intralipid
Intralipid
Normal Rat
Spleen Weight/Body Weight
% of Normal Rat Level
120
100
80
60
40
20
0
H&E
TUNEL
No Intralipid
Intralipid
Naïve
B
C
D
E
F
G
H
I
J
K
L
M
100 µm
50 µm
N
No Intralipid
Intralipid
Creatinine (µM)
250
200
150
100
50
0
0 hr
24 hr
48 hr
72 hr
O
No Intralipid
Intralipid
ALT (IU/L)
80
60
40
20
0
0 hr
24 hr
48 hr
72 hr

Fig S4.

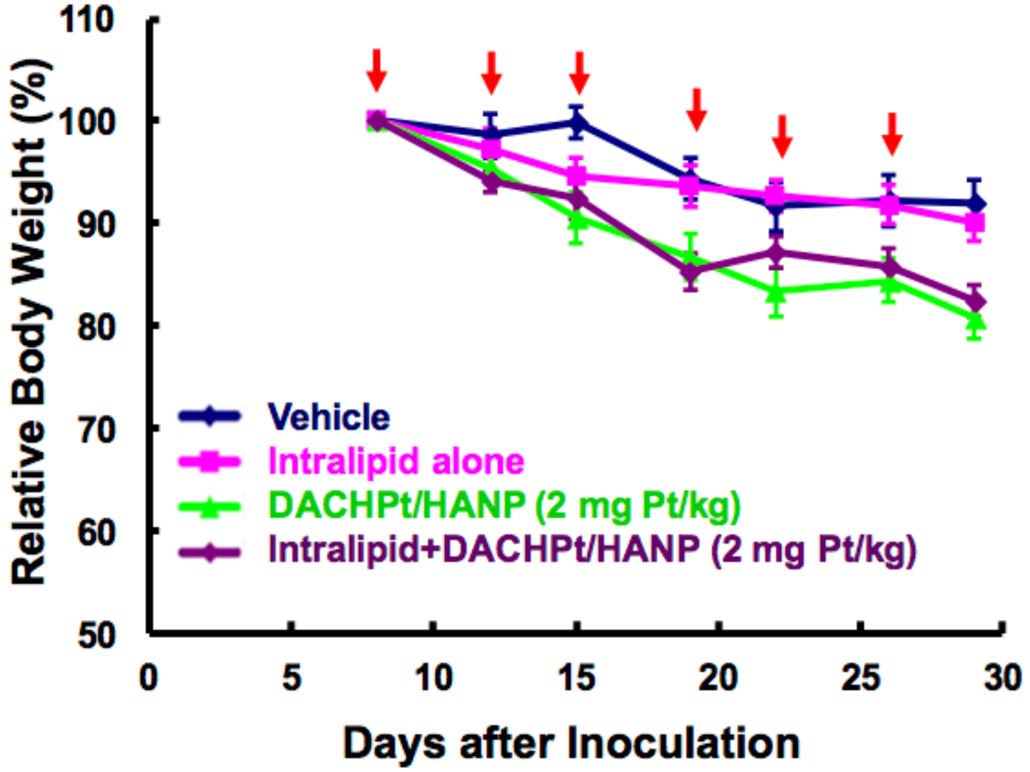
Relative Body Weight (%)
110
100
90
80
70
60
50
0
5
10
15
20
25
30
Days after Inoculation
Vehicle
Intralipid alone
DACHPt/HANP (2 mg Pt/kg)
Intralipid+DACHPt/HANP (2 mg Pt/kg)

Fig S5.
